# A candidate gene approach to study nematode resistance traits in naturally infected sheep

**DOI:** 10.1016/j.vetpar.2017.06.010

**Published:** 2017-08-30

**Authors:** Hazel Wilkie, Valentina Riggio, Oswald Matika, Louise Nicol, Kathryn A. Watt, Rona Sinclair, Alexandra M. Sparks, Daniel H. Nussey, Josephine M. Pemberton, Ross D. Houston, John Hopkins

**Affiliations:** a The Roslin Institute & R(D)SVS, University of Edinburgh, UK; b Institute of Evolutionary Biology, School of Biological Sciences, University of Edinburgh, UK

**Keywords:** *Teladorsagia circumcincta*, SNPs, *IL23R*, T cells, Parasite resistance, Sheep

## Abstract

Sheep naturally acquire a degree of resistant immunity to parasitic worm infection through repeated exposure. However, the immune response and clinical outcome vary greatly between animals. Genetic polymorphisms in genes integral to differential T helper cell polarization may contribute to variation in host response and disease outcome. A total of twelve single nucleotide polymorphisms (SNPs) were sequenced in *IL23R, RORC2* and *TBX21* from genomic DNA of Scottish Blackface lambs. Of the twelve SNPs, six were non-synonymous (missense), four were within the 3′ UTRs and two were intronic. The association between nine of these SNPs and the traits of body weight, faecal egg count (FEC) and relative *T. circumcincta* L3-specific IgA antibody levels was assessed in a population of domestic Scottish Blackface ewe lambs and a population of free-living Soay ewe lambs both naturally infected with a mixture of nematodes. There were no significant associations identified between any of the SNPs and phenotypes recorded in either of the populations after adjustment for multiple testing (Bonferroni corrected P value ≤ 0.002). In the Blackface lambs, there was a nominally significant association (P = 0.007) between *IL23R* p.V324M and weight at 20 weeks. This association may be worthy of further investigation in a larger sample of sheep.

## Highlights

• SNPs within genes associated with the development of nematode resistance were sequenced.
• The SNPs were segregating in two unrelated, domestic and feral, sheep breeds.
• More samples are required to determine significant associations between SNPs and traits.

## 1 Introduction

Young lambs are particularly susceptible to parasitic nematode infection, which is a major economic and welfare burden to sheep production (Wright, 2013). With repeated exposure, most lambs develop some level of protective immunity against the parasites (McRae et al., 2015). The use of genetic markers in selective breeding for resistant animals (currently selected based on lower faecal egg count (FEC), higher antibody (IgA) levels and higher weight compared to susceptible lambs; Beraldi et al., 2008) would allow for improved accuracy of selection and reduced need for routine phenotyping.

While genome wide association study (GWA) is a powerful method for detecting loci associated with the trait of interest, it requires a very large sample size to achieve adequate statistical power to detect loci with small to medium effects (Korte and Farlow, 2013). An alternative is the candidate gene approach in which the effects of polymorphisms within genes of relevance to the trait of interest are investigated (Brown et al., 2013). In the case of nematode resistance, candidate genes include those that are involved in the immune response to nematode infection.

Gene expression studies in sheep infected with *Teladorsagia circumcincta* have indicated that increased expression of inflammatory T helper type 1 (Th1) and/or Th17-assocatied genes correlate with nematode susceptibility (Gossner et al., 2012) while increased expression of Th2-associated genes correlate with resistance (Wilkie et al., 2016a, Wilkie et al., 2016b). During previous sequencing work (Nicol et al., 2016, Wilkie et al., 2016a) we identified potential SNPs within *TBX21* (expressed by Th1 cells)*, IL23R* and *RORC2* (expressed by Th17 cells). In the present study, these SNPs were sequenced and then tested for associations with nematode resistance and associated traits in naturally infected domestic Scottish Blackface and free-living Soay ewe lambs with recorded body weight, FEC and *T. circumcincta*-specific IgA levels (Davies et al., 2006, Riggio et al., 2013, Nussey et al., 2014). The two aims of the current study were (i) to confirm SNPs in the nematode resistance candidate genes *TBX21, IL23R* and *RORC2* in different sheep breeds, and (ii) to test for associations between these SNPs and traits related to nematode resistance in naturally infected populations.

## 2 Materials and methods

### 2.1 SNP identification and validation in the experimental Scottish Blackface lambs

Numerous potential SNPs were identified in the T helper cell transcription factor and cytokine receptor genes during previous expression and cDNA sequencing experiments (Nicol et al., 2016, Wilkie et al., 2016a). Twelve experimental Scottish Blackface lambs (described in full by Beraldi et al., 2008) were used to confirm the sequence of all potential SNPs. Genomic DNA was extracted from 20 mg abomasal lymph node tissue, preserved in RNAlater (Ambion, UK), using the Wizard^®^ SV Genomic DNA Purification System (Promega, UK) and was quantified by NanoDrop ND-1000 spectrophotometry; 200 ng was used as PCR template. FastStart Taq (Roche, UK) was used as per manufacturer’s instructions using the primers in Table S1. Six clones per SNP per lamb were sequenced as described previously (Wilkie et al., 2016a). A total of twelve SNPs in *TBX21, RORC2* and *IL23R* were thus confirmed using this method. After sequence confirmation it transpired that all twelve SNPs had already been identified in the dbSNP database (http://www.ncbi.nlm.nih.gov/projects/SNP/), therefore the SNP accession numbers (Table S1) were extracted.

### 2.2 Naturally infected Scottish Blackface population

DNA samples from Scottish Blackface ewe lambs (born between 2001 and 2003) previously analysed by Riggio et al. (2013), were used for SNP association analysis. Lambs were born on pasture and were continuously exposed to natural mixed nematode infection (including a number of genera such as *Teladorsagia, Trichostrongylus, Oesophagostomum, Chabertia, Bunostomum, Cooperia, Nematodirus* and *Haemonchus*). All lambs were sequentially sampled at 16, 20 and 24 weeks for strongyle (excluding *Nematodirus*) FEC and body weight; in an oversight, half of the 20-week-weight data was lost at the time of collection therefore only 116 lambs had weight data at this time point in the current study. Serum anti-*T. circumcincta* IgA levels were measured from blood samples collected when lambs were 24 weeks old.

### 2.3 Naturally infected soay population

DNA samples from feral Soay ewe lambs (born between 2002 and 2015) were also used for SNP association analysis; lambs with the highest and lowest FEC from each year were selected. In August, when lambs were between 16 and 20 weeks of age, around 60% of the resident population of Hirta, St Kilda, was rounded up in temporary corral traps; body weight was measured, faecal and blood samples were taken (Clutton-Brock and Pemberton, 2004). The Soay sheep were exposed to a mixture of gastro-intestinal nematodes including the genera *Teladorsagia, Trichostrongylus, Bunostomum, Chabertia, Trichuris, Capillaria* and *Nematodirus.*

### 2.4 Phenotypic analysis of naturally infected lambs

For all lambs in both populations, strongyle (excluding *Nematodirus*) FEC was obtained using a modified MacMaster Method and is reported as eggs per g faeces (epg) (Gulland and Fox, 1992, Riggio et al., 2013). Plasma fractions of the whole blood sample were used to determine serum anti-*T. circumcincta* IgA levels measured by ELISA (as described in Riggio et al., 2013 and Nussey et al., 2014) and expressed as: (sample OD – background OD)/(positive control OD – background OD) on each ELISA plate.

### 2.5 SNP genotyping allele frequencies and LD analysis

Genotyping was conducted by LGC Genomics using KASP™ technology (http://www.lgcgroup.com/products/kasp-genotyping-chemistry/). Genomic DNA was provided at 5 ng/μl per sample. Of the twelve SNPs identified, three did not pass quality control and were not analysed further.

For the nine SNPs genotyped in the two populations, Hardy Weinberg Equilibrium (HWE) was calculated and all SNPs were found to be in HWE (data not shown). A Chi Square test with 2 ° of freedom confirmed differences in genotype frequency between the populations (Table S2) using Graph Pad Prism v5 (Graph Pad software, USA).

The R^2^ between each SNP pair was estimated using Plink v1.07 (http://pngu.mgh.harvard.edu/∼purcell/plink/) (Purcell et al., 2007) to explore linkage disequilibrium (LD) within the Blackface and Soay populations.

### 2.6 Statistical analyses for SNP associations

A generalised linear model (GLM) in SAS 9.4 (2015) was used to explore the significance of the fixed effects. Significant effects were then fitted in a linear mixed model in ASReml (Gilmour et al., 2015) to estimate genetic parameters. The response variables were FEC, IgA and weight (at various ages in the Blackface lambs). The fixed effects fitted for the Blackface population were: early management group, lamb birth year, dam age at sampling (years) and litter size. The fixed effects fitted for the Soay population were: lamb birth year, lamb age at sampling (weeks), dam age at lambing (years) and litter size. In both cases animal was fitted as a random effect.

The animal models were fitted as follows:

y = Xb + Zu + e

Where y is a vector of observations on the specific trait; b is a vector of non-genetic fixed effects; u is direct additive genetic effects; X and Z are corresponding incidence matrices and e is the residual. The normal distributions assumed for the random effect were as follows: animal ∼N (0, Aσ^2^a), e ∼ N (0, Iσ^2^e), where A is the numerator relationship matrix, and I is an identity matrix of the order equal to the number of records. The relationship matrix was constructed using pedigree records of 960 Blackface sheep and 730 Soay sheep. A sire model (fitted as above but with sire as the random effect) was then used in the Blackface population to confirm associations. The SNPs were fitted as fixed effects in the association analyses.

Multiple testing was accounted for using a Bonferroni correction threshold of P ≤ 0.002. This was calculated as P = 0.05/(α × [β + γ]); where α corresponds to the (5) independent LD blocks of SNPs; β represents the (3) independent traits tested in the Soay lambs; γ corresponds to the (3) independent traits tested in the Blackface lambs (weight at different time points were correlated therefore were treated as one trait for correction purposes).

SNP effects were estimated as:

Additive effect, a = (AA − aa)/2;

Dominance effect, d = Aa − [(AA + aa)/2];

Percentage of additive variance (V_A_) due to the SNP = [2pq (a + d(p − q))2]/V_A_

where AA, Aa and aa were the predicted trait values for each genotype class; p and q were the allelic frequencies at the SNP locus. Animals with missing data were excluded from relevant analyses; the total number of lambs included in each analysis is detailed in Table S3. Statistical power was calculated using GWAPower software (Feng et al., 2011).

## 3 Results

### 3.1 SNP confirmation in Blackface lambs

A total of twelve SNPs in *IL23R, RORC2* and *TBX21* were verified by sequencing genomic DNA in experimental Blackface lambs (Table 1). Six SNPs were identified in *IL23R* on Chr 1 plus strand (NC_019458.2). Three SNPs (p.P110S, c.492-115A > G and c.492-102C > T) did not pass LGC Genomics quality control and were not analysed further (bold in Table 1). *IL23R* p.N287D was located in exon 7 within the extracellular domain. *IL23R* p.V324 M and *IL23R* p.K333N were in exon 8, between the ligand-binding motif and the transmembrane domain (Table 1). Four SNPs were identified in *RORC2,* on Chr 1 minus strand (NC_019458.2); *RORC2* p.E294Q was located in exon 5, between the predicted ligand and DNA-binding domains. *RORC2* p.A404T was situated in exon 8, within the ligand-binding domain. *RORC2* c.*25T > C and *RORC2* c.*109A > G were situated 25 and 109 nucleotides downstream of the stop codon respectively (Table 1). *TBX21* is located on ovine Chr 11 minus strand (NC_019468.2); *TBX21* c.*861A > G and *TBX21* c.*871A > G, both were located downstream of the stop codon within the predicted 3′ UTR (Table 1).

**Table 1:** Validation of SNP locations and effects in ovine *IL23R, RORC2* and *TBX21*.

SNP Accession No.^1^	Chr (strand)	Genome position^2^	Gene	Exon	cDNA position^3^	Protein position^4^	Effect
**rs595356447**	**1 (+)**	**42,469,152**	*IL23R*	**3**	**1292C > T**	**P110S**	**Missense**
rs408638389	1 (+)	42,499,077		7	1823A > G	N287D	Missense
rs426358915	1 (+)	42,512,431		8	1934G > A	V324M	Missense
rs405076951	1 (+)	42,512,460		8	1963A > T	K333N	Missense
**rs403830024**	**1 (+)**	**42,487,890**		**Intron 4–5**	**492-115A > G**		**Intron variant**
**rs415145731**	**1 (+)**	**42,487,903**		**Intron 4–5**	**492-102C > T**		**Intron variant**
rs159639535	1 (−)	100,654,917	*RORC2*	5	880G > C	E294Q	Missense
rs403822388	1 (−)	100,653,186		8	1210G > A	A404T	Missense
rs428174832	1 (−)	100,648,086		3′ UTR	*25T > C		Downstream gene variant
rs415026575	1 (−)	100,648,002		3′ UTR	*109A > G		Downstream gene variant
rs426434073	11 (−)	38,270,681	*TBX21*	3′ UTR	*861A > G		Downstream gene variant
rs411294999	11 (−)	38,270,671		3′ UTR	*871A > G		Downstream gene variant

1 SNP accession numbers from dbSNP database.

2 Genome position is nucleotide number in Oar v4.0.

3 cDNA position is relative to the ‘A’ of the start codon denoted as position 1.

4 Protein position indicates the amino acid residue affected. SNPs in **bold** were not sequenced in the naturally infected lambs. Chr; Chromosome.

### 3.2 Phenotype analysis of naturally infected populations

FEC data were natural log transformed (ln(epg + 15)) and IgA data were cube-root transformed for normal distributions, weight data were approximately normally distributed without transformation (Figs. S1 and S2). At all time points, mean FEC of the Blackface ewe lambs was lower than mean FEC in the Soay population (Fig. S3A) and mean Blackface body weight was greater than Soay mean body weight (Fig. S3B). Litter size was larger in the Blackface compared to the Soay population (Fig. S3C) and dams were older at lambing in the Soay compared to the Blackface (Fig. S3D).

### 3.3 SNP frequencies and LD analysis in naturally infected populations

Within each population, all SNPs were in HWE. The loci *RORC2* c.*25T > C, *RORC2* c.*109A > G and *TBX21* c.*861A > G were fixed in the Soay population (Table S2). Among those loci that segregated in both populations, there was a significant difference (P < 0.05) in allele frequencies at *IL23R* p.N287D, *IL23R* p.V324 M, *RORC2* p.E294Q and *TBX21* c*871A > G (Table S2).

In the Blackface population, *RORC2* c.*25T > C and *RORC2* c.*109A > G were in complete LD, whilst p.N287D and p.K333N in the *IL23R* gene had the second highest R^2^ value of 0.55 for this population (Fig. S4A). *IL23R* p.N287D and p.K333N were in complete LD in the Soay population, while *RORC2* p.E294Q and p.A404T had the second highest R^2^ value of 0.61 in this population (Fig. S4B). Lower R^2^ values were observed for other SNP pairs within genes. SNPs in different genes were not in LD.

### 3.4 SNP associations in naturally infected Scottish Blackface population

A statistical power calculation based on the sample size of the current study (n = 230) and assuming an R^2^ between marker and causative variant of 0.3 indicated that the study had a power of 0.82 to detect QTL explaining 5% of the phenotypic variation (h^2^ > 0.05).

**Fig. 1:**
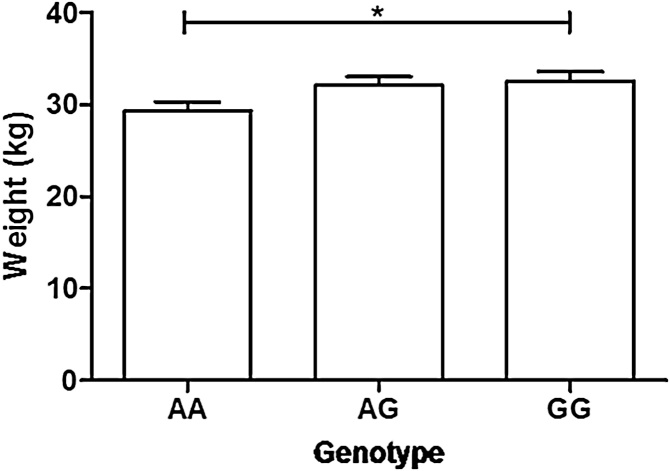
Model prediction of mean weight at 20 weeks based on *IL23R* p.V324 M allele in Blackface lambs. G allele encodes V; A allele encodes M. ^*^P value = 0.01. Error bars are SEM.

There were no significant (P ≤ 0.002) associations between the SNPs and the resistance traits in the Blackface lambs (Table S3). However, a nominally significant association (p = 0.007 and p = 0.004 fitting animal and sire model, respectively) was identified for *IL23R* p.V324 M and body weight at 20 weeks in this population (Table S3). The estimated additive genetic effect was 1.60 ± 0.51 kg (p = 0.002) with no significant dominance effects (0.60 ± 0.77 kg, p = 0.14). The percentage of additive variance (V_A_) due to the SNP was 6.41%. The heritability estimate for body weight at 20 weeks had a large point estimate but was not different from zero (0.72 ± 0.48), however, the predicted genotypic effects for weight at 20 weeks were significant (p = 0.01), with higher mean (±SEM) body weight in GG homozygotes (32.5 ± 1.1 kg) compared to AA homozygotes (29.3 ± 1.0 kg; Fig. 1).

### 3.5 SNP associations in naturally infected Soay population

There were so significant associations between any of the SNPs and phenotypes in the Soay population (Table S3). The association (P = 0.071) between IgA and *RORC2* p.A404T (Table S3) was the closest to the threshold but was not analysed further.

## 4 Discussion

This study identified SNPs within ovine *TBX21, RORC2* and *IL23R* genes in an experimentally challenged Scottish Blackface population. The SNPs were primarily situated within or in close proximity to functional domains of the genes, and it was hypothesised that they could result in altered gene function. This has been previously reported in human *IL23R* (Duerr et al., 2006) in which a SNP within *IL23R* was protective against inflammatory bowel disease (Di Meglio et al., 2011). Therefore, mutations within inflammation-associated genes can protect against inflammatory pathways potentially leading to increased Th2 and IgA responses (Langrish et al., 2004). The ovine SNPs within *IL23R, TBX21* and *RORC2* were therefore analysed for associations with FEC, IgA and body weight in two different naturally infected lamb populations. An advantage of the current study was the sequencing of the SNPs prior to association analysis to confirm that these SNPs represent real polymorphisms (Table 1) not predicted mutations. This study used two unrelated and phenotypically distinct (Fig. S3) sheep breeds indicating that the SNPs identified were not breed-specific. Functional analysis is required to confirm if these SNPs affect gene expression or protein structure and function as is hypothesised.

No significant associations were found between any of the SNPs and the traits considered in either of the populations. This is most likely due primarily to the small sample size and the polygenic nature of the traits (Sweeney et al., 2016).

In conclusion, this study identified and confirmed segregating SNP markers in genes of biological relevance to host immune response, and potentially nematode resistance. The nominally significant association between *IL23R* p.V324 M and body weight under infection indicates that this SNP may be worthy of further investigation in additional, larger sheep populations to test its association with nematode resistance.

## Conflict of interests

The authors declare that they have no conflicts of interest.
