# Supplementary material for: A candidate gene approach to study nematode resistance traits in naturally infected sheep
Source: Vet Parasitol. 2017 Aug 30;243:71–4. doi: 10.1016/j.vetpar.2017.06.010 (PMC5567408; doi:10.1016/j.vetpar.2017.06.010)
Supplement: Supplementary file 1 [file mmc1.pdf]

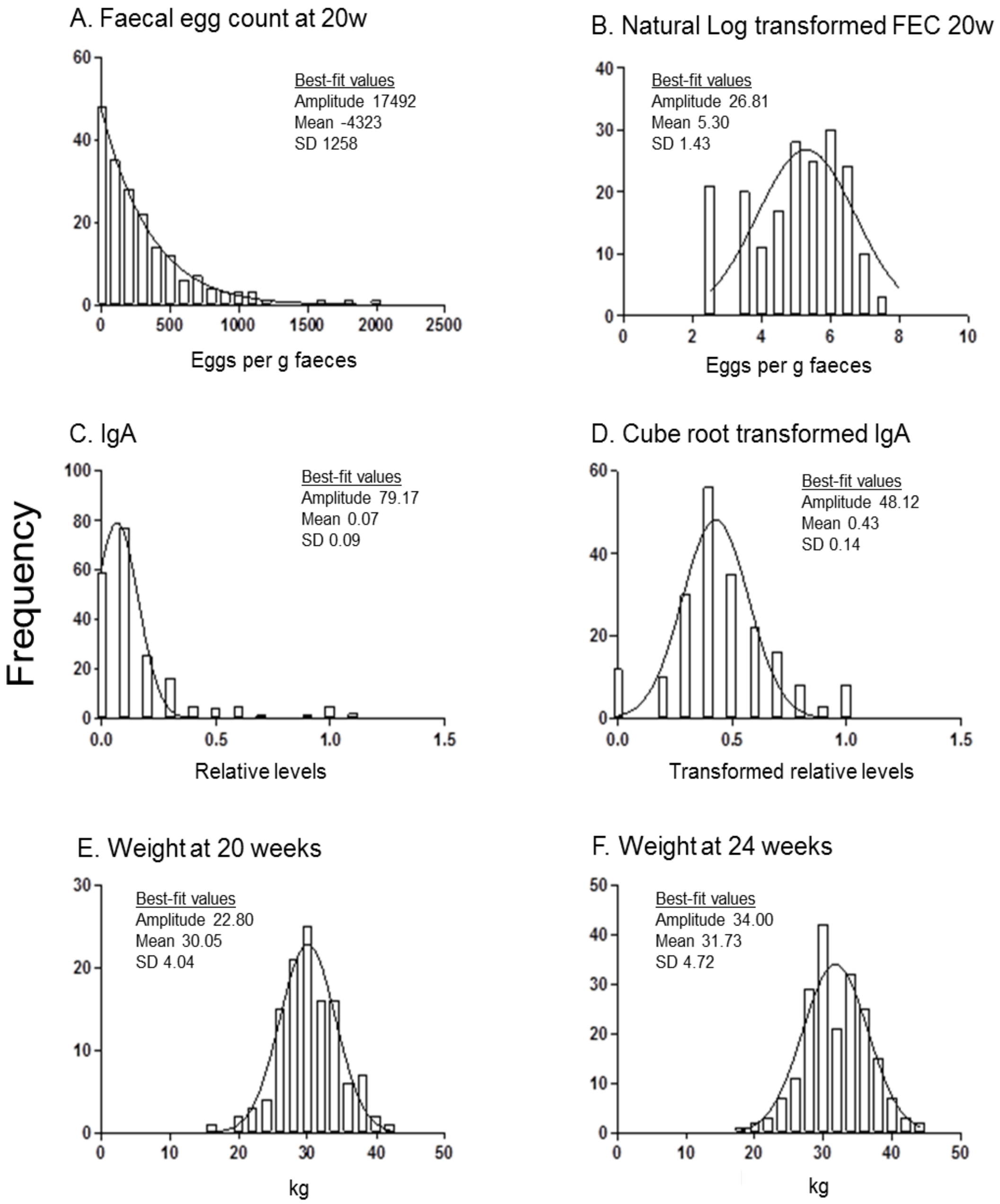

Figure S1 Distributions of phenotypes in Blackface population before and after normalisation. For SNP analysis, FEC was natural log transformed using ln(epg + 15) for normal distribution; IgA was cube root transformed for normal distribution; body weight was approximately normally distributed without transformation. Best-fit values of a Gaussian distribution (black line) analysis are displayed. FEC and Weight were recorded at age 16, 20 and 24 weeks for all lambs but only 20 week FEC (A and B) is displayed as an example to

indicate the distribution of the data; weight at 16 weeks was also normally distributed but is not shown. Graph Pad Prism v5 was used for histogram construction and analysis.
