# Supplementary material for: A candidate gene approach to study nematode resistance traits in naturally infected sheep
Source: Vet Parasitol. 2017 Aug 30;243:71–4. doi: 10.1016/j.vetpar.2017.06.010 (PMC5567408; doi:10.1016/j.vetpar.2017.06.010)
Supplement: Supplementary file 2 [file mmc2.pdf]

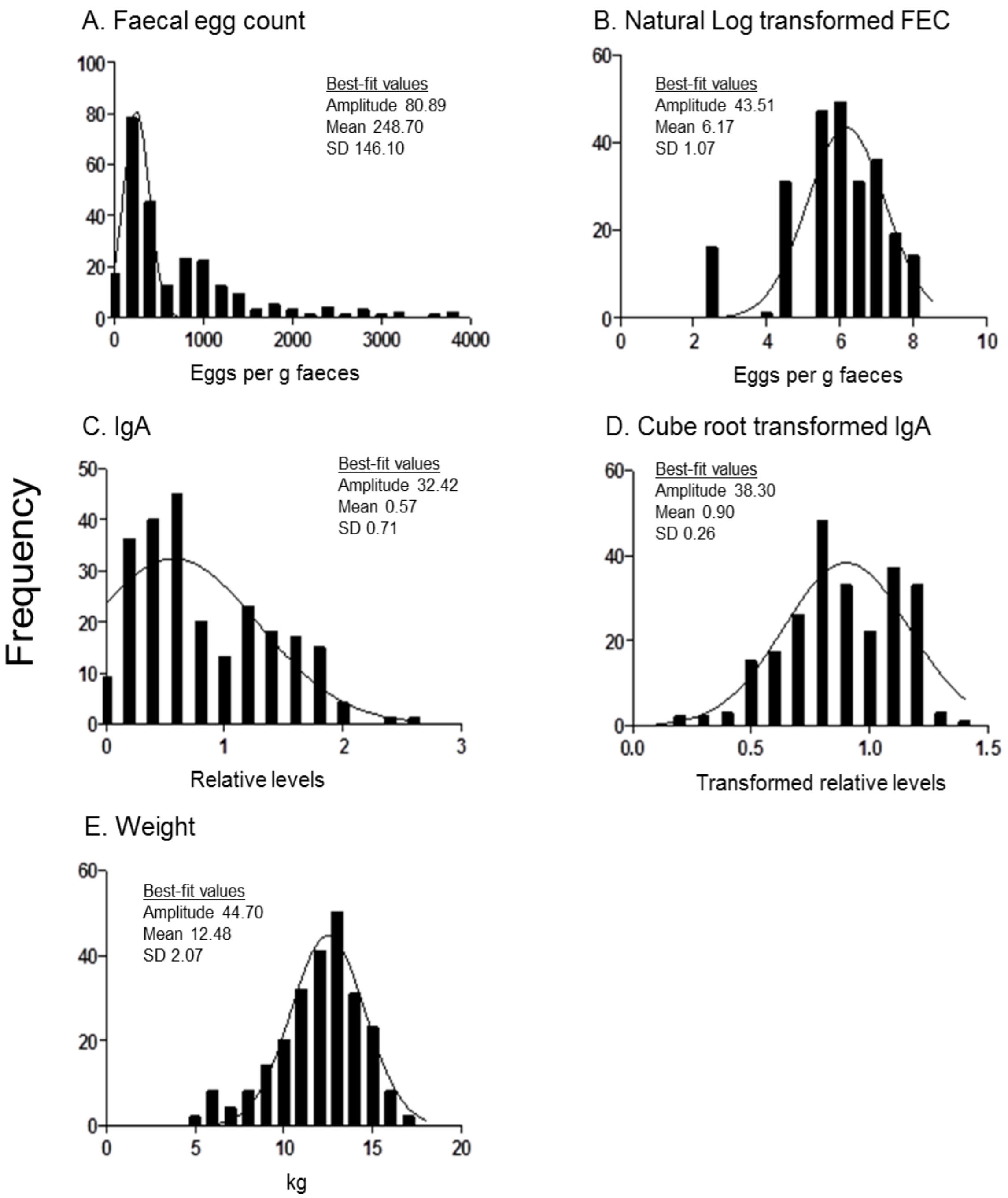

Figure S2 Distributions of phenotypes in Soay population before and after normalisation. For SNP analysis, FEC was natural log transformed using ln(epg + 15) for normal distribution; IgA was cube root transformed for normal distribution; body weight was approximately normally distributed without transformation. Best-fit values of a Gaussian distribution (black line) analysis are displayed. Lambs were on average 17 weeks old at data collection. Graph Pad Prism v5 was used for histogram construction and analysis.
