# Supplementary material for: A candidate gene approach to study nematode resistance traits in naturally infected sheep
Source: Vet Parasitol. 2017 Aug 30;243:71–4. doi: 10.1016/j.vetpar.2017.06.010 (PMC5567408; doi:10.1016/j.vetpar.2017.06.010)
Supplement: Supplementary file 3 [file mmc3.pdf]

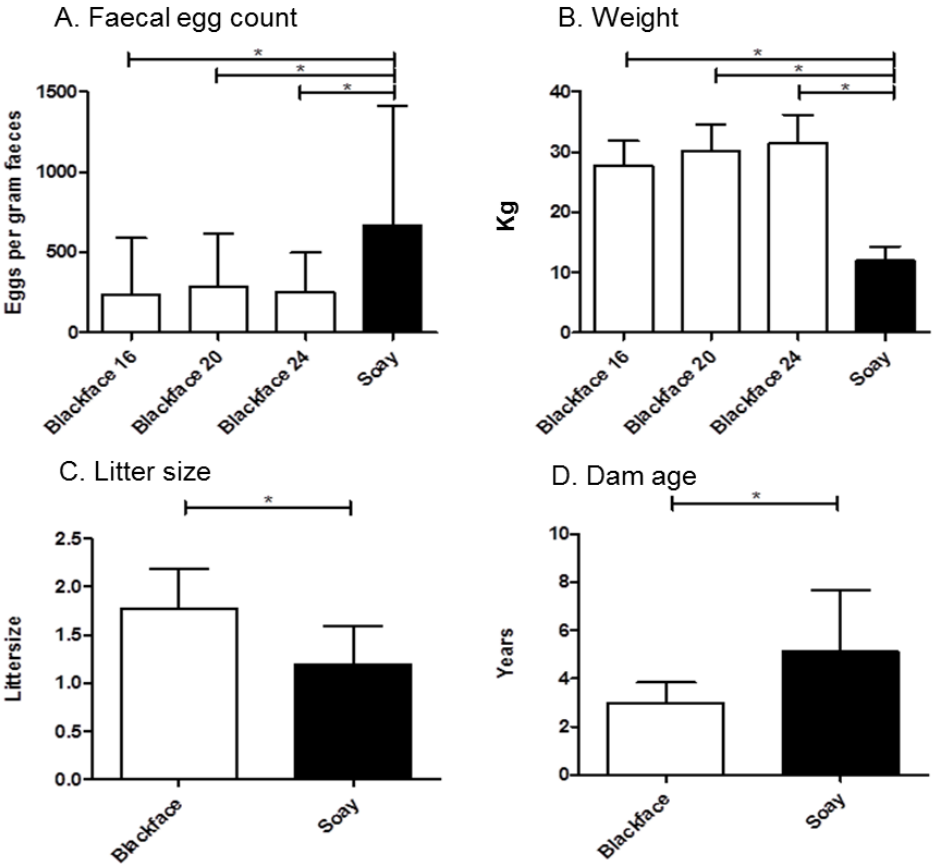

Figure S3 Phenotype summary of Blackface and Soay populations. Blackface data were collected when lambs were 16, 20 and 24 weeks old, Soay lambs were on average 17 weeks old at data collection. Graph Pad Prism version 5 was used for statistical analysis of the traits; Kruskal-Wallis ANOVA with Dunn’s multiple comparison test for FEC data, one-way ANOVA with Tukey’s post-hoc test for weight data, T test analysis of litter size and dam age at lambing. $P \leq 0.05$ was the threshold for significant differences. Error bars are ± SD. * P value < 0.001.
