# Supplementary material for: A candidate gene approach to study nematode resistance traits in naturally infected sheep
Source: Vet Parasitol. 2017 Aug 30;243:71–4. doi: 10.1016/j.vetpar.2017.06.010 (PMC5567408; doi:10.1016/j.vetpar.2017.06.010)
Supplement: Supplementary file 4 [file mmc4.pdf]

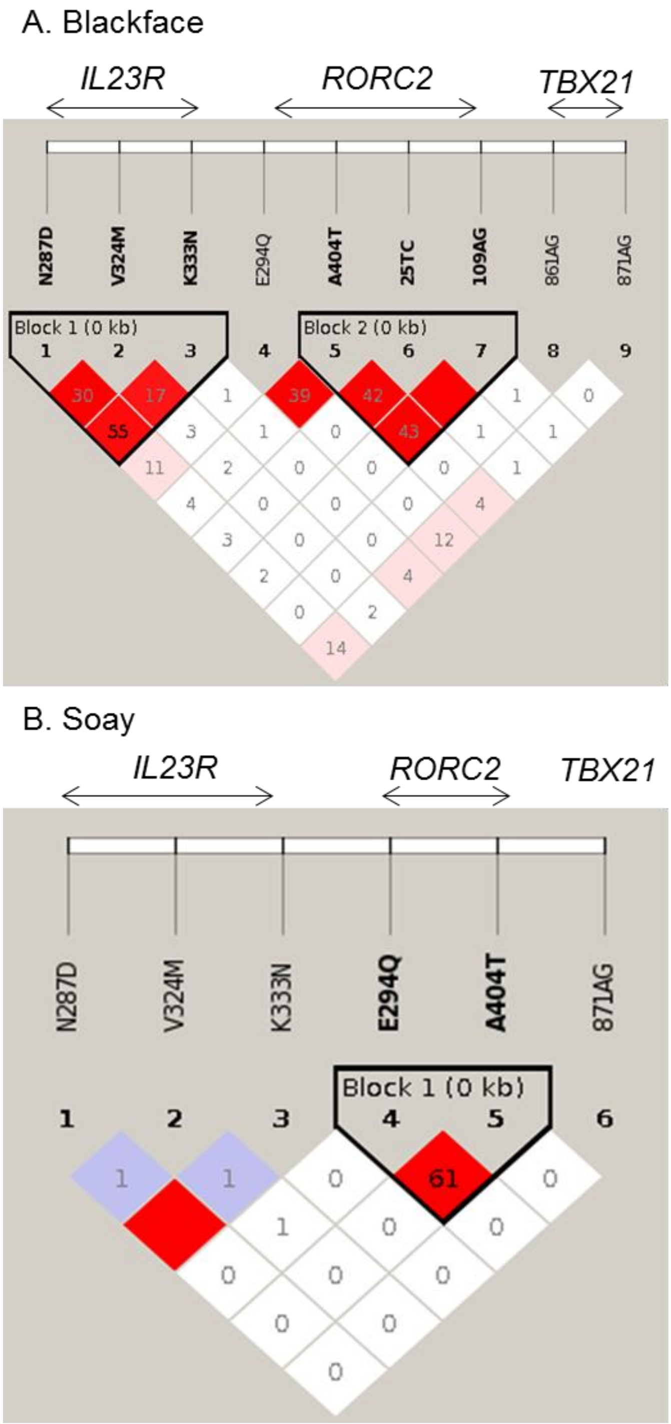

Figure S4 Pairwise LD heatmap analysis of SNPs in Blackface and Soay populations. SNPs are named based on their location within the coding region of the gene. Three SNPs were non-segregating in the Soays hence were not included in the LD analysis. SNPs with R^2 > 30% were assumed to be in LD; red boxes with no R^2 value indicate an LD of 100%; pink, white and blue boxes indicate below-threshold R^2 values.
