# Supplementary material for: A candidate gene approach to study nematode resistance traits in naturally infected sheep
Source: Vet Parasitol. 2017 Aug 30;243:71–4. doi: 10.1016/j.vetpar.2017.06.010 (PMC5567408; doi:10.1016/j.vetpar.2017.06.010)
Supplement: Supplementary file 5 [file mmc5.pdf]

Table S1 Primers for sequencing candidate gene regions for SNP discovery.

Gene	SNP Accession No.	SNP[1]	Forward Primer (5'-3')	Reverse Primer (5'-3')	Product Size
IL23R	**rs595356447**	**p.P110S**	CAACAGCTCGCCTTTGGTATAAC	ACCCCAACACTTACATCCAGAAG	134
	rs408638389	p.N287D	CCCAGGATTGAGAAGGGTCTAG	GGGTGAACTCCAGGGTTGC	258
	rs426358915	p.V324M	AAACTGGGCAACCTGAGTGC	ACCTAAGCAGGATTTTCTTACCAG	170
	rs405076951	p.K333N			
	**rs403830024**	**c.492-115A>G**	CACACCGTGACCCTGGATG	GACTTCATTTGATTAGGTCAGTGC	404
	**rs415145731**	**c.492-102C>T**			
RORC	rs159639535	p.E294Q	GCAGGGAGAACTTCTACGGC	TCTCCGTCAGGGAAGCATAAG	172
	rs403822388	p.A404T	GCTGGTCAGGATGTGCCG	GAACAGCTCCACGCCACC	82
	rs428174832	c.*25T>C	TGCGGAGCCTGTGTAGCC	AGGCCGGTGGGCTGAAAC	294
	rs415026575	c.*109A>G			
TBX21	rs426434073	c.*861A>G	CAGTCATATACCTGGTGCTGC	TCTGGACACCACTCTCTGTA	857
	rs411294999	c.*871A>G			

[1]SNPs are named according to the amino acid residue they alter (for missense SNPs) or according to their location in the coding region (for intronic and 3' UTR SNPs). PCR parameters were: 95°C for 4 min, 35 cycles of 95°C for 15 sec, 60°C for 20 sec, 72°C for 30 sec, final elongation of 72°C for 7 min. Products were cloned and sequenced as described previously (Wilkie *et al.*, 2016a); six clones per animal per SNP were sequenced using T7 and SP6 primers. SNPs highlighted **bold** could not be sequenced in the naturally infected populations.
