# Supplementary material for: A candidate gene approach to study nematode resistance traits in naturally infected sheep
Source: Vet Parasitol. 2017 Aug 30;243:71–4. doi: 10.1016/j.vetpar.2017.06.010 (PMC5567408; doi:10.1016/j.vetpar.2017.06.010)
Supplement: Supplementary file 6 [file mmc6.pdf]

Table S2 SNP allele frequencies in Blackface and Soay populations.

SNP[1]	SNP Accession No.	Blackface		Soay		X^2 (P value)[2]
		**P (major allele)**	**Q (minor allele)**	**P (major allele)**	**Q (minor allele)**	
IL23R p.N287D	rs408638389	0.78 (A)	0.22 (G)	0.85 (A)	0.15 (G)	**6.97 (0.03)**
IL23R p.V324M	rs426358915	0.52 (A)	0.48 (G)	0.91 (G)	0.09 (A)	**155.9 (<0.0001)**
IL23R p.K333N	rs405076951	0.85 (T)	0.15 (A)	0.84 (T)	0.16 (A)	1.24 (0.53)
RORC2 p.E294Q	rs159639535	0.76 (G)	0.24 (C)	0.64 (G)	0.36 (C)	**15.55 (0.0004)**
RORC2 p.A404T	rs403822388	0.56 (A)	0.44 (G)	0.52 (A)	0.48 (G)	1.33 (0.51)
RORC2 c.*25T>C	rs428174832	0.75 (C)	0.25 (T)	1.00 (C)		
RORC2 c.*109A>G	rs415026575	0.75 (G)	0.25 (A)	1.00 (G)		
TBX21 c.*861A>G	rs426434073	0.96 (A)	0.04 (G)	1.00 (A)		
TBX21 c.*871A>G	rs411294999	0.68 (A)	0.32 (G)	0.70 (G)	0.30 (A)	**110.5 (<0.0001)**

[1]SNPs are named according to the amino acid residue they alter (for missense SNPs) or according to their location in the coding region (for intronic and 3' UTR SNPs). [2]P values from a test of heterogeneity between populations on the counts of the three genotypes at each locus, using a Chi Square Test with 2 degrees of freedom. All SNPs were in HWE within the populations (Chi square test, df = 1, P values not shown). $P \leq 0.05$ was the threshold for significant differences. Significant P values in **bold**.
