# Supplementary material for: A candidate gene approach to study nematode resistance traits in naturally infected sheep
Source: Vet Parasitol. 2017 Aug 30;243:71–4. doi: 10.1016/j.vetpar.2017.06.010 (PMC5567408; doi:10.1016/j.vetpar.2017.06.010)
Supplement: Supplementary file 7 [file mmc7.pdf]

Table S3 Table of P values of associations between all genotyped SNPs and phenotypes in Blackface and Soay populations.

	Phenotype	No. lambs	*IL23R*			*RORC2*				*TBX21*	
		included[1]	N287D	V324M	K333N	E294Q	A404T	*25T>C	*109A>G	*861A>G	*871A>G
BLACKFACE	FEC 16	183	0.932	0.701	0.978	0.617	0.457	0.590	0.664	0.524	0.351
	FEC 20	183	0.796	0.739	0.620	0.408	0.602	0.369	0.393	0.501	0.848
	FEC 24	195	0.581	0.446	0.900	0.964	0.646	0.750	0.746	0.136	0.638
	Weight 16	187	0.940	0.082	0.865	0.118	0.931	0.952	0.873	0.727	0.357
	Weight 20	116	0.413	**0.007**			0.447	0.895	0.838		
	Weight 24	193	0.870	0.041	0.266	0.495	0.893	0.723	0.665	0.332	0.443
	IgA	193	0.946	0.276	0.849	0.087	0.105	0.189	0.250	0.468	0.762
SOAY	FEC	244	0.345	0.778	0.332	0.802	0.878				0.679
	Weight	243	0.778	0.710	0.664	0.314	0.698				0.321
	IgA	242	0.153	0.674	0.133	0.296	0.071				0.387

[1]Lambs with missing phenotype data were excluded from relevant analyses therefore the number of lamb records analysed per trait is detailed. SNPs are named according to the amino acid residue they alter (for missense SNPs) or according to their location in the coding region (for 3' UTR SNPs). IgA (relative levels) were collected when Soay lambs were 16-20 weeks old and Blackface lambs were 24 weeks old. **Bold**: Nominally significant p value. Blanks indicate insufficient data for analysis.
